# A Classification of Speakers

**Published:** 1899-05

**Authors:** William Whitford

**Affiliations:** Chicago


### A CLASSIFICATION OF SPEAKERS.

By WILLIAM WHITFORD, Chicago.

A STUDY of the different styles of speakers, encountered during an extensive and varied reportorial experience, may be considered an education in itself, and by virtue of his vocation the shorthand writer is compelled to study them. As there is no one more competent to critise speakers than the man upon whom devolves the duty of reporting them, I have made an attempt to classify the various speakers whose utterances I have reported from time to time in medical societies. What pertains to physicians as speakers will apply equally well to preachers, political speakers, etc.

We have: (1) The moderately slow speaker. (2) The exuberant and tempestous speaker. (3) The musical, flowery speaker. (4) The loud, husky speaker, whose voice is somewhat indistinct. (5) The grandiloquent speaker. (6) The rapid and spasmodic speaker. (7) The excessively rapid speaker. (8) The rapid, involved and indistinct speaker. (9) The man who hurls disconnected sentences at the stenographer. (10) The clear, distinct, unassuming speaker, who talks with absolute precision, with perfect grammar. He is a rara avis, although stenographers occasionally meet him. (11) The man who commences his speech in a deliberate, measured, distinct, far-reaching tone of voice, and who, when he becomes influenced by the magnetism of his audience and their rapt attention, gives vent to rare flights of oratory. (12) The irrepressible speaker, who does not know enough to sit down while he is down, but who monopolises the precious time of an important convention in saying practically nothing; still his remarks are reported, transcribed and then perhaps liberally blue-penciled, or consigned by the secretary of the association or editor to the waste-basket. (13) The man who jumbles up his nominative, his accusative and his verb with such picturesque incoherence that it is almost impossible to make head or tail of what he means. (14) The man who threads his way through a long and apparently intricate sentence, never losing the connection of the parts, and coming out at the end with absolute precision. (15) The man who does not realise that his first duty is to make himself heard. (16) The man who never completes his sentences, but is utterly oblivious of that fact when he critises the report of his speech. (17) The man who misquotes, and then blames the shorthand writer if he has not supplied the correct quotation. (18) The foreigner, who imagines he speaks like a native. (19) The man who drops his voice toward the end of sentences. (20) The low, mumbling conversational style of speaker, who indulges in a free and rapid flow of words which taxes the reporter's skill and dexterity. (21) The man who is extremely subtle in the use of words and phrases. The shades of meaning are so delicate by the judicious selection of them that the stenographer must retain the original force and color of each sentence. (22) The sympathetic, lachrymose speaker. (23) The man who habitually clothes his hazy ideas in loose, disjointed talk, which, if transcribed as uttered, would mar, if not ruin, his reputation. (24) The man with a slow and fatiguing mode of utterance, whose sentences are diffuse, intricate and deficient, both in eloquence and precision. (25) The man who is clear, forcible, persuasive, with an eloquence natural and direct. This type of speaker usually spends much time in meditation and in the composition of his speeches. (26) The man whose speeches are notable for their subtlety of discrimination and depth of learning. (27) The man who evolves original thoughts, whose imagination pictures vivid scenes, and whose voice and gesture emphasise living truths. (28) The eccentric speaker, who stifles and distorts natural eloquence. His introductory is long; divisions and sub-divisions are perhaps illustrated by a pointless story. (29) The man who carefully selects his words and utters sentences that are models of syntax. (30) The man whose style is natural, easy and varied, with short clauses expressing vivid ideas. (31) The tautological speaker. (32) The vivid, brilliant speaker, who pours out a torrent of words, dexterously handled, and whose remarks are full of sudden turns and surprises, but are illumined by ingenious reasonings. (33) The man with a strange and unfamiliar style, whose sentences are portentously long. (34) The man who speaks rapidly and monotonously, becomes entangled in his own sentences and mispronounces words. (35) The man who is known for his elegant diction, his scholarly references and the ornateness of his phraseology. (36) The man who stutters, speaks indistinctly and yet is extremely technical. (37) The pompous speaker.—*The Chicago Clinic.*

